# Genetic islands in pome fruit pathogenic and non-pathogenic *Erwinia* species and related plasmids

**DOI:** 10.3389/fmicb.2015.00874

**Published:** 2015-08-28

**Authors:** Pablo Llop

**Affiliations:** Department of Evolutionary Genetics, Cavanilles Institute, University of Valencia, Paterna, Valencia, Spain

**Keywords:** *Erwinia* genus, gene similarity, transfer elements, genetic diversity, gene interaction

## Abstract

New pathogenic bacteria belonging to the genus *Erwinia* associated with pome fruit trees (*Erwinia, E. piriflorinigrans, E. uzenensis*) have been increasingly described in the last years, and comparative analyses have found that all these species share several genetic characteristics. Studies at different level (whole genome comparison, virulence genes, plasmid content, etc.) show a high intraspecies homogeneity (i.e., among *E. amylovora* strains) and also abundant similarities appear between the different *Erwinia* species: presence of plasmids of similar size in the pathogenic species; high similarity in several genes associated with exopolysaccharide production and hence, with virulence, as well as in some other genes, in the chromosomes. Many genetic similarities have been observed also among some of the plasmids (and genomes) from the pathogenic species and *E. tasmaniensis* or *E. billingiae*, two epiphytic species on the same hosts. The amount of genetic material shared in this genus varies from individual genes to clusters, genomic islands and genetic material that even may constitute a whole plasmid. Recent research on evolution of erwinias point out the horizontal transfer acquisition of some genomic islands that were subsequently lost in some species and several pathogenic traits that are still present. How this common material has been obtained and is efficiently maintained in different species belonging to the same genus sharing a common ecological niche provides an idea of the origin and evolution of the pathogenic *Erwinia* and the interaction with non-pathogenic species present in the same niche, and the role of the genes that are conserved in all of them.

## Introduction

The genus *Erwinia* belongs to the *Enterobacteriaceae* family and essentially comprises plant-associated bacteria that are pathogenic and epiphytic to pome fruit trees (Palacio-Bielsa et al., 2011). The most important species is *Erwinia amylovora*, causal agent of fire blight on rosaceous hosts, which is present worldwide and produces very high economic losses (Bonn and van der Zwet, 2000). Other pathogenic *Erwinia* species have been described in the last decades: *Erwinia pyrifoliae*, a pathogen described in Asian pear isolated in South Korea (Kim et al., 1999; Rhim et al., 1999; Shrestha et al., 2003); *E. piriflorinigrans*, isolated in 1999 in Spain, causes necrosis of pear blossoms (López et al., 2011), and *Erwinia uzenensis* from Japan, which produces bacterial black shoot disease (BBSDP) on European pear (Matsuura et al., 2012). Other related *Erwinia* species, *E. tasmaniensis* and *E. billingiae*, are epiphytes in the same hosts. All these species are genetically and phenotypically closely related, although they can be distinguished by taxonomic criteria (Mergaert et al., 1999; Kube et al., 2008; Palacio-Bielsa et al., 2011).

In the last years, several sequencing projects have been carried out which included all the *Erwinia* species except *E. uzenensis*. All have provided interesting clues about the relationships among these organisms and the exchange of genetic material (Sundin, 2007; Kube et al., 2008, 2010; Sebaihia et al., 2010; Smits et al., 2010a,b; Powney et al., 2011; Smits et al., 2013; Ismail et al., 2014; Smits et al., 2014). Because *E. amylovora* is a very important pathogen, the majority of information is related to it. Genetic studies have divided *E. amylovora* strains into two major groups with different host range: strains isolated from *Spiraeoideae* and from *Rosoideae* (*Rubus* spp., Mann et al., 2013). The genomes of *Spiraeoideae*-infecting strains are highly homogeneous, and greater diversity was observed between *Spiraeoideae*- and *Rubus*-infecting strains, the majority of which was attributed to variable genomic islands (Triplett et al., 2006; Smits et al., 2010b).

Comparative genomics of *E. amylovora* strains from different origins showed that the pan-genome is highly conserved relative to other phytopathogenic bacteria species, with homogeneity of 99.99% identity at the nucleotide level (Smits et al., 2010b; Zhao and Qi, 2011; Mann et al., 2013). The genomes of two *E. pyrifoliae* strains sequenced (Ep1/96) and DSM 12163 (Ep16/99) are almost identical whereas the two saprophytic species are distantly related to the pathogenic species, with *E. tasmaniensis* more related than *E. billingae* (Kube et al., 2010; Smits et al., 2010a). Comparison of genomes of Japanese (Ejp617) and Korean (Ep1/96) *E. pyrifoliae* strains revealed a high level of genome conservation (more than 95% nucleotide sequence identity) despite the numerous insertion/deletion rearrangements and inversions associated with Insertion Sequences (IS). The differences are mainly based on transposases, phage-related genes, and single genes (Smits et al., 2010a; Thapa et al., 2013). The genes acquired by horizontal gene transfer (HGT) are introduced via mobile genetic elements (MGEs) and incorporated into the chromosome by homologous or illegitimate recombination.

Other characteristics observed are related to the genome size. Differences in size between *E. pyrifoliae* and *E. tasmaniensis* genomes are due to the insertion of MGEs in the *E. pyrifoliae* genome that code transposases, integrases, and phage-related proteins. The prevalence of a high number of mobile elements in Ep1/96 may suggest frequent genomic changes and a higher rate of evolution (Smits et al., 2010a; Thapa et al., 2013).

Ancestral origins of several virulence factors have been found, and the two major virulence determinants required for *E. amylovora* to infect and cause disease are the genes involved in amylovoran biosynthesis and the type III secretion systems (T3SS). Other genes that could have been acquired after a divergence of pathogenic species are flagellar genes (Zhao et al., 2011) and the type VI secretion systems (T6SS; Smits et al., 2011; Mann et al., 2013). In this review, I will discuss the presence of transfer elements, involving from individual genes to entire plasmids, and how these genetic transfers intervene in the emergence of characteristics like pathogenicity, virulence and the fitness of the pome fruit erwinias (Zhao and Qi, 2011).

## Exopolysaccharide Biosynthesis

Exopolysaccharide (EPS) is a pathogenicity factor contributing to biofilm formation of *E. amylovora* (Koczan et al., 2009), encoded by the *ams* operon. This gene cluster is present in the genomes of *E. amylovora*, *E. pyrifoliae*, and *E. piriflorinigrans* (Bernhard et al., 1996; Kube et al., 2008). In the sequenced *E. tasmaniensis* and *E. billingiae* genomes these genes are present in a different cluster (*cps*) yielding an EPS more related to stewartan of *Pantoea stewartii* subsp. *stewartii* (Coplin et al., 1996; Kim et al., 2002; Smits et al., 2010a; Malnoy et al., 2012). The hypothesis could be that an *Erwinia* ancestor produced an EPS similar to stewartan of *P. stewartii* (Coplin et al., 1996; Kube et al., 2008), and the differentiation took place at or after the separation of the pathogenic *Erwinia* from *E. tasmaniensis*, and this would indicate that the genes involved in amylovoran production are probably acquired (Bernhard et al., 1996; Smits et al., 2011).

## Type III Secretion Systems

The T3SS is a protein complex found in several Gram-negative bacteria with a needle-like structure used as a sensory probe to detect the presence of eukaryotic organisms and to secrete and inject virulence factors into the host cells (Galan and Collmer, 1999) and are located in pathogenicity islands (PAIs) integrated into the genomes in the plant pathogens (Oh et al., 2005; Toth et al., 2006). The PAI in all isolates analyzed of *E. amylovora* is divided into four distinct DNA regions, and is delimited by genes suggesting horizontal gene transfer and the remnants of an integrative conjugative element (ICE) are present at the flank of the Hrp cluster (Wei and Beer, 1993; Oh and Beer, 2005; Oh et al., 2005; Mann et al., 2012, 2013; Vrancken et al., 2013). The Hrp region is conserved in the *Spiraeoideae* strains sequenced (CFBP 1430, ATCC 49946; Smits et al., 2010b) whereas the genomes of several *Rubus* strains (ATCC BAA-2158, Ea644, and MR-1; Powney et al., 2011) showed larger sizes. Similarly, the island transfer (IT) regions of *Spiraeoideae*-infecting strains of *E. amylovora* (IT: group of MGEs that reside in a host chromosome but retain the ability to excise and to transfer by conjugation) are highly conserved (>99% nucleotide identity and identical synteny), but the IT regions of the *Rubus*-infecting strains all vary in sequence identity and length. Comparative genome sequences revealed two additional T3SS PAIs (PAI2 and PAI3) and two flagellar T3SS systems (Fla1 and Fla2; Zhao et al., 2009; Smits et al., 2011; Zhao and Qi, 2011). PAI2 and PAI3 have a significantly lower G+C content and are closely related to those of *Sodalis glossinidius* (an endosymbiont of the tsetse fly) and to the pathogens *Salmonella* and *Yersinia* (Dale et al., 2001; Young and Young, 2002; Gendlina et al., 2007; Zhao et al., 2009; Smits et al., 2010b). Sequences upstream of PAI2 and PAI3 contained genes associated with MGEs, thus, the insertion of a mobile element deleted a part of PAI-2 in *E. tasmaniensis* Et1/99 (Kube et al., 2008), and PAI2 is lost in *E. pyrifoliae* (Smits et al., 2010a). It could be speculated that *E. amylovora* may have acquired these novel T3SS PAIs from other bacteria associated with their insect vectors during evolution, or that these novel T3SS PAIs may contribute to the association of *E. amylovora* with its insect vectors (Zhao and Qi, 2011). Two other sets of genes encoding for flagellar assembly and chemotaxis related proteins were found in the genome of *E. pyrifoliae*. One set is tightly clustered and the encoded proteins show high identity with the corresponding proteins of *Salmonella* and *Escherichia* spp. (Kube et al., 2008). This suggests that the entire region was acquired as a genomic island via horizontal genetic transfer (Smits et al., 2010a).

## Type VI Secretion Systems

**FIGURE 1:**
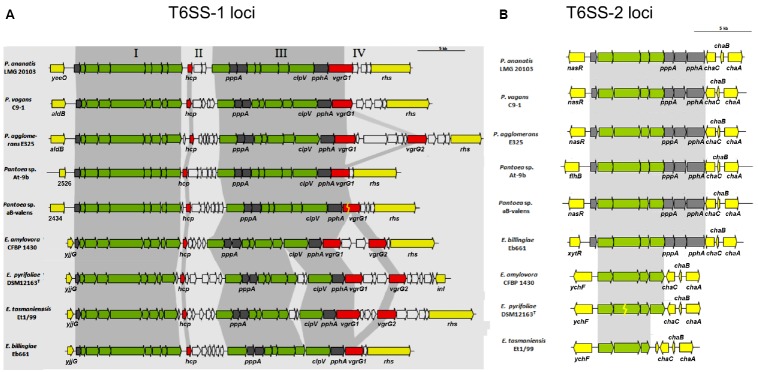
**The orthologous T6SS-1 (A) and T6SS-2 (B) loci in ***Pantoea*** and ***Erwinia*** species. (A)** The conserved regions (block I and III) are shaded in gray, while the non-conserved hcp and vgrG islands are not shaded. Genes encoding conserved domain proteins identified by Boyer et al. (2009) are represented by green arrows, and gray arrows indicate other genes conserved among the *Pantoea* and *Erwinia* T6SS-1 loci which were not identified as part of the conserved core described by Boyer et al. (2009). Red arrows represent the hcp and vgrG genes while genes not conserved among the *Pantoea* and *Erwinia* species are colored in white. **(B)** The orthologous T6SS-2 loci in *Pantoea* and *Erwinia* species. Genes encoding proteins with the conserved domains identified by Boyer et al. (2009) are represented by green arrows while the gray arrows indicate other genes conserved among the *Pantoea* and *Erwinia* T6SS-2 loci, which were not identified as part of the conserved core. White arrows represent the genes not conserved among the *Pantoea* and *Erwinia* species (Illustration from De Maayer et al., 2011).

Type VI secretion systems (T6SS) have been identified in at least a quarter of the sequenced Gram-negative bacteria (Boyer et al., 2009; Records, 2011), and three gene clusters (T6SS1-3) have been found in the genome of *E. amylovora* CFBP 1430 (Smits et al., 2010b). Comparison of the T6SS clusters among *Erwinia* and *Pantoea* species has identified conserved core regions and variable islands (De Maayer et al., 2011). T6SS clusters 1 and 2 are highly similar to *E. pyrifoliae* DSM 12163T and *E. tasmaniensis* Et1/99, with the exception of some genes encoding hypothetical proteins that do not belong to the core genes of T6SS (Bingle et al., 2008). *E. amylovora* showed variations within non-conserved islands of T6SS-1 in regions that share high sequence similarity to bacteria of the genus *Pantoea*, including the plant pathogen *Pantoea stewartii* subsp. *stewartii* (Braun, 1982; Smits et al., 2010a; Figures 1A,B). The third T6SS cluster was identified only in *E. amylovora* CFBP 1430, located within a putative genomic island and, therefore, might be acquired by horizontal gene transfer (Smits et al., 2010b).

## Gene Transfer in Related Plasmids in *Erwinia* spp

One of the most obvious differences among strains of *Erwinia* spp is the presence of different plasmids in all genome-sequenced *Erwinia* spp. New plasmids have been described during the latest genome sequencing projects, and is the largest factor influencing the pan-genome size of *E. amylovora* (McGhee et al., 2002; Maxson-Stein et al., 2003; Foster et al., 2004; Sebaihia et al., 2010; Powney et al., 2011; Kamber et al., 2012; Llop et al., 2012; Ismail et al., 2014). The analyses performed have revealed a strong relationship with other plant and human pathogenic and non-pathogenic bacteria, and constitute the widest host range of the genetic exchange in *Erwinia* spp. Some of the genetic material exchanged involving different plasmids are described below.

## Streptomycin Resistance in pEA34

Streptomycin resistance (SmR) *E. amylovora* strains were found harboring transposon Tn*5393* including the *strA-strB* gene pair (Chiou and Jones, 1993; McGhee and Sundin, 2011). Tn*5393* was introduced to *E. amylovora* on the conjugative plasmid pEa34. This transposon is also present in *Pantoea agglomerans* and is thought to have been transferred to *E. amylovora* on pEA34 (Chiou and Jones, 1991). Plasmid pEA34 could have originated from the insertion of Tn*5393* into a 28 kb plasmid no related with pEA29 (Chiou and Jones, 1993; Jones and Schnabel, 2000), and the transposon probably moved from pEa34 to pEA29 because this transposon was found in pEA29 (Burr et al., 1988; Chiou and Jones, 1993; McManus and Jones, 1994; Sundin and Bender, 1995, 1996; McGhee and Sundin, 2011). An additional insertion of Tn*5393* was found into the *thiO* gene of pEA29 (McGhee and Jones, 2000).

Other *E. amylovora* isolates were found harboring genes *strA-strB* within the same Tn*5393* transposon in a different plasmid (pEA8.7; Sundin and Bender, 1996). This plasmid is identical to the SmR plasmid RSF1010 that has a broad distribution among enteric bacteria and also encodes the *strA-strB* gene pair (Palmer et al., 1997). These genes are identical to the streptomycin resistance genes found in at least 14 genera of gram-negative animal and human pathogens. In the clinical strains, the genes reside on small broad-host-range plasmids like RSF1010 and pBP1 or large self-transmissible R plasmids like pGS05 and pCJ004 (Sundin and Bender, 1996; McGhee and Sundin, 2011).

## Plasmids and Genetic Transfer Elements

*Erwinia amylovora* isolates from Poland and Belgium showed the presence of a new plasmid of 68 kb (pEA68). It is closely related to other plasmids from different *E. amylovora* strains, pEA72 from USA (Sebaihia et al., 2010) and pEA78 in *E. amylovora* from Mexico (Smits et al., 2014). The amino acid sequence identity between these plasmids range between 60 and 90% for the CDS shared, and include genes of transfer and mobilization (*mob*, *tra*, and *trb*). Large regions between the *repA* gene and the *mobABC* gene cluster are divergent in all three plasmids, indicating that these regions are highly variable likely due to horizontal gene transfer (Ismail et al., 2014).

**FIGURE 2:**
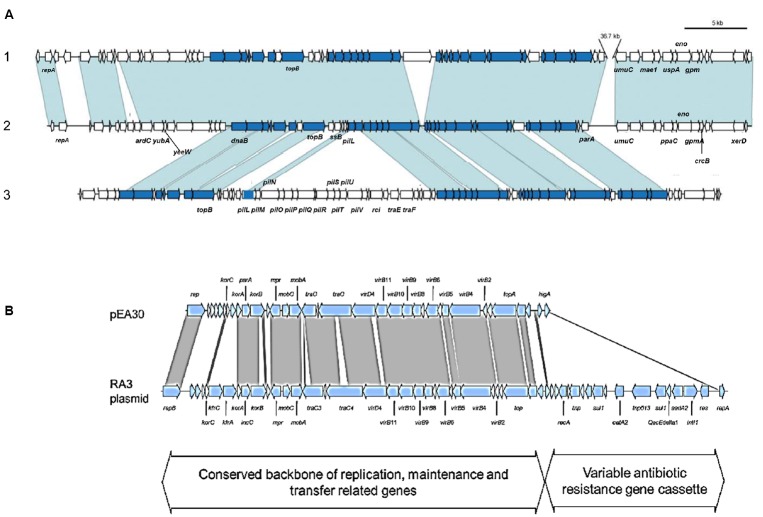
**(A)** Comparison of plasmid pEB102 of *E. billingiae* Eb661 (1) with *E. amylovora* ACW 56400 plasmid pEI70 (2), and the conserved region of GAI-2 of *Pectobacterium atrosepticum* SCRI 1043 (3). Orthologous genes are indicated by blue shading (conserved ICE element genes) and shading. Genes in white do not have orthologs in these regions (Illustration from Llop et al., 2011). **(B)** Comparison of plasmid pEA30 of CFBP 2585 (Ea495) to the RA3 plasmid of *Aeromonas hydrophila*. The RA3 plasmid is the archetype of the IncU plasmids which are a distinct group of mobile elements with highly conserved backbones and variable antibiotic resistance gene cassettes. Conservation between pEA30 and RA3 (represented by the gray shaded lines) is limited to the conserved backbone of replication, maintenance and transfer related genes. Nucleotide similarity searches to known sequences in GenBank indicate that pEA30 has 70% total sequence coverage and 64–81% identity of all high-scoring segment pair matches related to the RA3 plasmid (Illustration from Mann et al., 2013).

Plasmid pEI70 was found in Spanish strains of *E. amylovora* (Llop et al., 2006). It is conjugative and widespread in European countries, and similarly to pEA29, it induces an increase in symptoms development but with no specific pathogenicity genes (Llop et al., 2008, 2011). pEI70 is almost entirely included in plasmid pEB102 from *E. billingiae*, with nucleotide sequence identities superior to 98%. The organization is identical as well, with only a 36-kb region in pEB102 absent in pEI70. Another major feature of pEI70 is the presence of an Integrating Conjugative Element (ICE) that shares similarities to specific regions of *Pseudomonas fluorescens* Pf-5 (Mavrodi et al., 2009) and *Pectobacterium atrosepticum* SCRI 1043 (Toth et al., 2006; Llop et al., 2011; Figure 2A) and its possible role in the fitness of the bacteria (Llop et al., 2011).

Plasmid pEL60 was reported in *E. amylovora* strains from Lebanon in half of the isolates analyzed. pEL60 has strong relationships with other enterobacterial plasmids, with a high similarity to the *Citrobacter freundii* plasmid pCTX-M3, sharing 66 of the 68 ORFs it contains (Foster et al., 2004). Transfer genes are similar to genes on plasmid pACM1 from *Klebsiella oxytoca* (Preston et al., 2000) and to *tra* genes from plasmids isolated from other enteric bacteria and from the plant pathogen *P. syringae* pv. *tomato* DC3000. These observations suggest that the environmental enteric plant pathogen *E. amylovora* can access the horizontal gene pool shared among clinical enteric bacteria.

In *E. pyrifoliae* strains, plasmids pEP36 and pEJ30 were found in South Korean and Japan isolates, respectively. They are nearly identical, both contain the element IS285 and the transposon (Tn*5394*) is missing in pEJ30. Other significant similarities of pEP36 were found in *Yersinia pestis* genome and *Shigella flexneri* SHI-2 PAIs (Mann et al., 2013). In *E. piriflorinigrans* strains, sequencing of a common 37-kb plasmid (pEPIR37) revealed high similarity to plasmids pEA29 of *E. amylovora*, and plasmids pEP36 and pEJ30 of *E. pyrifoliae* (McGhee et al., 2002; Maxson-Stein et al., 2003; Barbé et al., 2012). The replication origin has high homology with plasmids pET46 of *E. tasmaniensis* and pPag3 of *P. vagans* (Kube et al., 2008; Smits et al., 2010c) but also, a fragment of RepA protein (94% sequence identity) present in pEA29, pEJ30, and pEP36 plasmids, is present in pEPIR37. 12 CDS are similar to genes present in the genomes of *E. pyrifoliae*, *E. tasmaniensis*, and *E. billingiae* (Barbé et al., 2012).

A novel plasmid pEA30 was found in *E. amylovora* strain CFBP 2585, and nucleotide searches showed that is closely related to the RA3 plasmid of *Aeromonas hydrophila* (64–81% identity). Its high genetic similarity to RA3, a broad host range and self-transmissible plasmid, stably maintained in *Alpha*-, *Beta*-, and *Gammaproteobacteria*, is another example of the possible generation of the mobilome in pome fruit erwinias by means of plasmids present in the environment (Kulinska et al., 2008; Mann et al., 2013; Figure 2B).

In the plasmid pEA29 from *E. amylovora*, apart from the similarities with plasmids pEP36, pEJ30, and pEPIR37, remnants of several IS detected resembled insertion elements identified previously in other unrelated bacteria. A vestige of Tn*2501* and direct repeats found in the IS911 were detected in all derivatives of pEA29 (McGhee and Jones, 2000). A 108 aa CDS with similarity to ParA from *Agrobacterium tumefaciens* was also found (Kim and Geider, 1999). The presence of a partial *parA* gene may explain the occurrence of the 8-bp repeats found consistently in this plasmid.

An striking feature of *E. pyrifoliae* strain Ep1/96 is the presence of an assumed non-ribosomal peptide synthetase EppT (NRPS) with high similarity with a protein of unknown function from *Photorhabdus luminescens* (syn. *Xenorhabdus luminescens*), an enterobacterial pathogen of insects, and an NRPS of the distantly related soft rot pathogen *P. atrosepticum* encoded in an island typical for horizontal gene transfer (Duchaud et al., 2003; Bell et al., 2004; Kube et al., 2010). Similar NRPS proteins are also encoded in the genome of *E. tasmaniensis* strain Et1/99 and *E. billingiae* strain Eb661, but differ in size and domain content. The presence of the *eppT* gene in this strain may be a result of an integration event as indicated by a phage integrase located upstream (Kube et al., 2010).

## Discussion

Many genomic studies on almost all the pome fruit *Erwinia* species performed in the last years have unveiled the occurrence of transposition events related to HGT, and the presence of different genetic elements. They have allowed inferring the evolution and relatedness of the species within this genus and offer broad information about the mobilome of the plant host erwinias, both pathogenic and epiphytic (Smits et al., 2011). The pathogenic species show the acquisition of a large range of pathogenicity and virulence factors and a reduction of chromosome size by a significant gene loss. The important pathogenicity factor EPS, is present only in the genomes of *E. amylovora*, *E. pyrifoliae*, and *E. piriflorinigrans* (Smits et al., 2010b, 2011; Kamber et al., 2012). All these features demonstrate that horizontal gene transfer is responsible for many of the differential features between these species and may have led to the emergence of pathogenic species from the non-pathogenic (Smits et al., 2011). It is interesting to point out that the largest factor accounting for the genetic variability influencing the pan-genome of this genus is due to the presence of plasmids (Smits et al., 2011; Malnoy et al., 2012). Plasmid sequencing has uncovered the relationship observed in some of the medium and small plasmids in *Erwinia* species. Thus, partial sequences of plasmids present in some *E. amylovora* strains are most probably originated from other plasmids from human and animal pathogens (plasmids pEA30 and RA3, Figure 2B) and also the high genetic identity between plasmids pEI70 from *E. amylovora* and pEB102 from the epiphytic species *E. billingiae* indicates that lateral transfer of almost entire extrachromosomal material could take place between species sharing host and niche and be stably maintained (Llop et al., 2011; Figure 2A). Several of the plasmids reported show the potential for conjugal transfer, such as pEL60 and pEI70 from *E. amylovora*, pEB170 of *E. billingiae*, and several plasmids from *E. tasmaniensis*, and others carry *mob* genes and may contain an *oriT* to be mobilized by Tra proteins of other plasmids (plasmids pEP05 and pEt46 from *E. pyrifoliae* and *E. tasmaniensis*, Kube et al., 2010). The chromosome of *E. tasmaniensis* Et1/99 encodes part of the central region of *E. pyrifoliae* plasmid pEP36 carrying the *thiOSGF* and the *betB* genes, but not the entire plasmid (Smits et al., 2010a). The most recent sequencing projects performed are still unveiling new plasmids that are related to other plasmids or/and genomes from other genera, indicating that in this genus the main genetic source of variability is this extrachromosomal material (Smits et al., 2011, 2014; Ismail et al., 2014). Then, as reflected in this review, the mobilome in this genus shows an extended presence of transferable elements of different sizes, affecting all the species and situated mainly in PAIs related to virulence determinants, but also to the fitness of the bacterium, and all this information allows the knowledge about how the plant pathogens of the *Erwinia* pome fruits could access the gene pools of other enteric bacteria through horizontal transfer. As special features of the HGT in this genus are the role of MGEs based on plasmids in the acquisition of new traits related not only on classical aspects (i.e., antibiotic resistance) but in bacterial fitness that leads to a better survival in epiphytic species or increased aggressiveness in pathogenic ones. Also, the spread and stability of entire plasmids in both pathogenic and non-pathogenic species is remarkable. Many different factors can influence the prevalence of HGT in a community, but we do not know how the composition of the community shapes the likelihood of HGT events. In this case, knowledge of the bacterial community composition of the different species that are present in a particular site would be of great interest because HGT might be easier in a community composed of closely related species, and some species seem to be more prone to HGT events than others are. This would provide useful information for illuminating patterns of gene transfer in the microbial world.

### Conflict of Interest Statement

The author declares that the research was conducted in the absence of any commercial or financial relationships that could be construed as a potential conflict of interest.

## References

[B1] Barbé S. Llop P. Blom J. Cabrefiga J. Goesmann A. Duffy B. (2012). Complete sequence of *Erwinia piriflorinigrans* plasmids pEPIR37 and pEPIR5 and role of pEPIR37 in pathogen virulence. Plant Pathol. 62, 786–798. 10.1111/ppa.12002

[B2] Bell K. S. Sebaihia M. Pritchard L. Holden M. T. Hyman L. J. Holeva M. C. (2004). Genome sequence of the enterobacterial phytopathogen *Erwinia carotovora* subsp. atroseptica and characterization of virulence factors. Proc. Natl. Acad. Sci. U.S.A. 101, 11105–11110. 10.1073/pnas.0402424101 15263089 PMC503747

[B3] Bernhard F. Schullerus D. Bellemann P. Geider K. Nimtz M. Majerczak D. R. (1996). Genetics and complementation of DNA regions involved in amylovoran synthesis of *Erwinia amylovora* and stewartan synthesis of *Erwinia stewartii*. Acta Hortic. 411, 269–274. 10.17660/actahortic.1996.411.53

[B4] Bingle L. E. H. Bailey C. M. Pallen M. J. (2008). Type VI secretion: a beginner’s guide. Curr. Opin. Microbiol. 11, 3–8. 10.1016/j.mib.2008.01.006 18289922

[B5] Bonn W. G. van der Zwet T. (2000). Distribution and Economic Importance of Fire Blight. Fire Blight: the Disease and its Causative Agent, Erwinia amylovora. London: CABI Publishing 37–53 10.1079/9780851992945.0037

[B6] Boyer F. Fichant G. Berthod J. Vandenbrouck Y. Attree I. (2009). Dissecting the bacterial type VI secretion system by a genome wide in silico analysis: what can be learned from available microbial genomic resources? BMC Genomics 10:104–111. 10.1186/1471-2164-10-104 19284603 PMC2660368

[B7] Braun E. J. (1982). Ultrastructural investigation of resistant and susceptible maize inbreeds infected with *Erwinia stewartii*. Phytopathology 72, 159–166. 10.1094/Phyto-72-159

[B8] Burr T. J. Norelli J. L. Katz B. Wilcox W. F. Hoying S. A. (1988). Streptomycin resistance of *Pseudomonas syringae* pv. *papulans* in apple orchards and its association with a conjugative plasmid. Phytopathology 78, 410–413. 10.1094/Phyto-78-410

[B9] Chiou C. S. Jones A. L. (1991). The analysis of plasmid-mediated streptomycin resistance in *Erwinia amylovora*. Phytopathology 81, 710–714. 10.1094/Phyto-81-710

[B10] Chiou C. S. Jones A. L. (1993). Nucleotide sequence analysis of a transposon (Tn5393) carrying streptomycin resistance genes in *Erwinia amylovora* and other gram-negative bacteria. J. Bacteriol. 175, 732–740. 8380801 10.1128/jb.175.3.732-740.1993 PMC196212

[B11] Coplin D. L. Majerczak D. R. Bugert P. Geider K. (1996). Nucleotide sequence analysis of the *Erwinia stewartii* cps gene cluster for synthesis of stewartan and comparison to the *Erwinia amylovora* ams cluster for synthesis of amylovoran. Acta Hortic. 411, 251–5717. 10.17660/actahortic.1996.411.49

[B12] Dale C. Young S. A. Haydon D. T. Welburn S. C. (2001). The insect endosymbiont *Sodalis glossinidius* utilizes a type III secretion system for cell invasion. Proc. Natl. Acad. Sci. U.S.A. 98, 1883–1888. 10.1073/pnas.98.4.1883 11172045 PMC29351

[B13] De Maayer P. Venter S. N. Kamber T. Duffy B. Coutinho T. A. Smits T. H. M. (2011). Comparative genomics of the type VI secretion systems of Pantoea and *Erwinia* species reveals the presence of putative effector islands that may be translocated by the VgrG and Hcp proteins. BMC Genomics 12:576. 10.1186/1471-2164-12-576 22115407 PMC3235180

[B14] Duchaud E. Rusniok C. Frangeul L. Buchrieser C. Givaudan A. Taourit S. (2003). The genome sequence of the entomopathogenic bacterium *Photorhabdus luminescens*. Nat. Biotechnol. 21, 1307–1313. 10.1038/nbt886 14528314

[B15] Foster G. C. McGhee G. C. Jones A. L. Sundin G. W. (2004). Nucleotide sequences, genetic organization, and distribution of pEU30 and pEL60 from *Erwinia amylovora*. Appl. Environ. Microbiol. 70, 7539–7544. 10.1128/AEM.70.12.7539-7544.2004 15574957 PMC535195

[B16] Galan J. E. Collmer A. (1999). Type III secretion machines: bacterial devices for protein delivery into host cells. Science 284, 1322–1328. 10.1126/science.284.5418.1322 10334981

[B17] Gendlina I. Held K. G. Bartra S. S. Gallis B. M. Doneanu C. E. Goodlett D. R. (2007). Identification and type III-dependent secretion of the *Yersinia pestis* insecticidal-like proteins. Mol. Microbiol. 64, 1214–1227. 10.1111/j.1365-2958.2007.05729.x 17542916

[B18] Ismail E. Blom J. Bultreys A. Ivanovic M. Obradovic A. van Doorn J. (2014). A novel plasmid pEA68 of *Erwinia amylovora* and the description of a new family of plasmids. Arch. Microbiol. 196, 891–899. 10.1007/s00203-014-1028-5 25178659

[B19] Jones A. L. Schnabel E. L. (2000). “The development of streptomycin resistant strains of *Erwinia amylovora*,” in Fire Blight: the Disease and its Causative Agent Erwinia amylovora, ed. Vanneste J. L. (Wallingford: CAB International), 235–251. 10.1079/9780851992945.0235

[B20] Kamber T. Smits T. H. M. Rezzonico F. Duffy B. (2012). Genomics and current genetic understanding of *Erwinia amylovora* and the fire blight antagonist *Pantoea vagans*. Trees Struct. Funct. 26, 227–238. 10.1007/s00468-011-0619-x

[B21] Kim W. S. Geider K. (1999). Analysis of variable short-sequence DNA repeats on the 29 kb plasmid of *Erwinia amylovora* strains. Eur. J. Plant Pathol. 105, 703–713. 10.1023/A:1008723717211

[B22] Kim W. S. Gardan L. Rhim S. L. Geider K. (1999). *Erwinia pyrifoliae* sp. nov., a novel pathogen that affects Asian pear trees (*Pyrus pyrifolia* Nakai). Int. J. Syst. Bacteriol. 49, 899–906. 10.1099/00207713-49-2-899 10319516

[B23] Kim W. S. Schollmeyer M. Nimtz M. Wray V. Geider K. (2002). Genetics of biosynthesis and structure of the capsular exopolysaccharide from the Asian pear pathogen *Erwinia pyrifoliae*. Microbiology 148, 4015–4024. 12480905 10.1099/00221287-148-12-4015

[B24] Koczan J. M. McGrath M. J. Zhao Y. Sundin G. W. (2009). Contribution of *Erwinia amylovora* exopolysaccharides amylovoran and levan to biofilm formation: implications in pathogenicity. Phytopathology 99, 1237–44. 10.1094/PHYTO-99-11-1237 19821727

[B25] Kube M. Migdoll A. M. Gehring I. Heitmann K. Mayer Y. Kuhl H. (2010). Genome comparison of the epiphytic bacteria *Erwinia billingiae* and *E. tasmaniensis* with the pear pathogen *E. pyrifoliae*. BMC Genomics 11:393. 10.1186/1471-2164-11-393 20565991 PMC2897811

[B26] Kube M. Migdoll A. M. Müller I. Kuhl H. Beck A. Reinhardt R. (2008). The genome of *Erwinia tasmaniensis* strain Et1/99, a non-pathogenic bacterium in the genus *Erwinia*. Environ. Microbiology 10, 2211–2222. 10.1111/j.1462-2920.2008.01639.x 18462403

[B27] Kulinska A. Czeredys M. Hayes F. Jagura-Burdzy G. (2008). Genomic and functional characterization of the modular broad-host-range RA3 plasmid, the archetype of the IncU group. Appl. Environ. Microbiol. 74, 4119–4132. 10.1128/AEM.00229-08 18502921 PMC2446526

[B28] Llop P. Barbé S. López M. M. (2012). Functions and origin of plasmids in *Erwinia* species that are pathogenic to or epiphytically associated with pome fruit trees. Trees Struct. Funct. 26, 31–46. 10.1007/s00468-011-0630-2 25983394 PMC4425259

[B29] Llop P. Cabrefiga J. Smits T. H. M. Dreo T. Barbé S. Pulawska J. (2011). *Erwinia amylovora* novel plasmid pEI70: complete sequence, biogeography, and role in aggressiveness in the fire blight phytopathogen. PLoS ONE 6:e28651. 10.1371/journal.pone.0028651 22174857 PMC3235134

[B30] Llop P. Donat V. Rodríguez M. Cabrefiga J. Ruz L. Palomo J. L. (2006). An indigenous virulent strain of *Erwinia amylovora* lacking the ubiquitous plasmid pEA29. Phytopathology 96, 900–907. 10.1094/PHYTO-96-0900 18943756

[B31] Llop P. González R. Pulawska J. Bultreys A. Dreo T. López M. M. (2008). The new plasmid pEI70 is present in *Erwinia amylovora* European strains. Acta Hortic. 793, 131–136. 10.17660/actahortic.2008.793.15

[B32] López M. M. Roselló M. Llop P. Ferrer S. Christen R. Gardan L. (2011). *Erwinia piriflorinigrans* sp. nov., a novel pathogen that causes necrosis of pear blossoms. Int. J. Syst. Evol. Microbiol. 61, 561–567. 10.1099/ijs.0.020479-0 20382791

[B33] Malnoy M. Martens S. Norelli J. L. Barny M. Sundin G. W. Smits T. H. M. (2012). Fire Blight: applied genomic insights of the pathogen and host. Ann. Rev. Phytopathol. 50, 475–494. 10.1146/annurev-phyto-081211-172931 22702352

[B34] Mann R. A. Blom J. Bühlmann A. Plummer K. M. Beer S. V. Luck J. E. (2012). Comparative analysis of the Hrp pathogenicity island of *Rubus*- and *Spiraeoideae*-infecting *Erwinia amylovora* strains identifies the IT region as a remnant of an integrative conjugative element. Gene 504, 6–12. 10.1016/j.gene.2012.05.002 22579880

[B35] Mann R. A. Smits T. H. M. Bühlmann A. Blom J. Goesmann A. Frey J. E. (2013). Comparative genomics of 12 strains of *Erwinia amylovora* identifies a pan-genome with a large conserved core. PLoS ONE 8:e55644. 10.1371/journal.pone.0055644 23409014 PMC3567147

[B36] Matsuura T. Mizuno A. Tsukamoto T. Shimizu Y. Saito N. Sato S. (2012). *Erwinia uzenensis* sp. nov., a novel pathogen that affects European pear trees (*Pyrus communis* L.) Int. J. Syst. Evol. Microbiol. 62, 1799–1803. 10.1099/ijs.0.032011-0 21986725

[B37] Mavrodi D. V. Loper J. E. Paulsen I. T. Thomashow L. S. (2009). Mobile genetic elements in the genome of the beneficial rhizobacterium *Pseudomonas fluorescens* Pf-5. BMC Microbiol. 9:8. 10.1186/1471-2180-9-8 19144133 PMC2647930

[B38] Maxson-Stein K. McGhee G. C. Smith J. J. Jones A. L. Sundin G. W. (2003). Genetic analysis of a pathogenic *Erwinia* sp. isolated from pear in Japan. Phytopathology 93, 1393–1399. 10.1094/PHYTO.2003.93.11.1393 18944067

[B39] McGhee G. C. Jones A. L. (2000). Complete nucleotide sequence of ubiquitous plasmid pEA29 from *Erwinia amylovora* strain Ea88: gene organization and intraspecies variation. Appl. Environ. Microbiol. 66, 4897–4907. 10.1128/AEM.66.11.4897-4907.2000 11055941 PMC92397

[B40] McGhee G. C. Schnabel E. L. Maxson-Stein K Jones B. Stromberg V. K. Lacy G. H. (2002). Relatedness of chromosomal and plasmid DNAs of *Erwinia pyrifoliae* and *Erwinia amylovora*. Appl. Environ. Microbiol. 68, 6182–6192. 10.1128/AEM.68.12.6182-6192.2002 12450843 PMC134437

[B41] McGhee G. C. Sundin G. W. (2011). Evaluation of kasugamycin for fire blight management, effect on nontarget bacteria, and assessment of kasugamycin resistance potential in *Erwinia amylovora*. Phytopathology 101, 192–204. 10.1094/PHYTO-04-10-0128 20923369

[B42] McManus P. S. Jones A. L. (1994). Epidemiology and genetic analysis of streptomycin-resistant *Erwinia amylovora* from Michigan and evaluation of oxytetracycline for control. Phytopathology 84, 627–633. 10.1094/Phyto-84-627

[B43] Mergaert J. Hauben L. Cnockaert M. C. Swings J. (1999). Reclassification of non-pigmented *Erwinia herbicola* strains from trees as *Erwinia billingiae* sp. nov. Int. J. Syst. Bacteriol. 49, 377–383. 10.1099/00207713-49-2-377 10319458

[B44] Oh C-S. Beer S. V. (2005). Molecular genetics of *Erwinia amylovora* involved in the development of fire blight. FEMS Microbiol. Lett. 253, 185–192. 10.1016/j.femsle.2005.09.051 16253442

[B45] Oh C. -S. Kim J. F. Beer S. V. (2005). The Hrp pathogenicity island of *Erwinia amylovora* and the identification of three novel genes required for systemic infection. Mol. Plant Pathol. 6, 125–138. 10.1111/j.1364-3703.2005.00269.x 20565644

[B46] Palacio-Bielsa A. Roselló M. Llop P. López M. M. (2011). *Erwinia* spp. from pome fruit trees: similarities and differences among pathogenic and non-pathogenic species. Trees Struct. Funct. 26, 13–29. 10.1007/s00468-011-0644-9

[B47] Palmer E. L. Teviotdale B. L. Jones A. L. (1997). A relative of the broad-host-range plasmid RSF1010 detected in *Erwinia amylovora*. Appl. Environ. Microbiol. 63, 4604–4607. 9361446 10.1128/aem.63.11.4604-4607.1997 PMC168779

[B48] Powney R. Smits T. H. M. Sawbridge T. Frey B. Blom J. Frey J. E. (2011). Genome sequence of an *Erwinia amylovora* strain with restricted pathogenicity to *Rubus* plants. J. Bacteriol. 193, 785–786. 10.1128/JB.01352-10 21131493 PMC3021219

[B49] Preston K. E. Radomski C. C. A. Venezia R. A. (2000). Nucleotide sequence of a 7-kb fragment of pACM1 encoding an IncM DNA primase and other putative proteins associated with conjugation. Plasmid 44, 12–23. 10.1006/plas.2000.1472 10873523

[B50] Records A. R. (2011). The type VI secretion system: a multi-purpose delivery system with a phage-like machinery. Mol Plant Microbe Interact. 24, 751–757. 10.1094/MPMI-11-10-0262 21361789

[B51] Rhim S. L. Völksch B. Gardan L. Paulin J. P. Langlotz C. Kim W. S. (1999). *Erwinia pyrifoliae*, an *Erwinia* species different from *Erwinia amylovora*, causes a necrotic disease of Asian pear trees. Plant Pathol. 48, 514–520. 10.1046/j.1365-3059.1999.00376.x

[B52] Sebaihia M. Bocsanczy A. M. Biehl B. S. Quail M. A. Perna N. T. Glasner J. D. (2010). Complete genome sequence of the plant pathogen *Erwinia amylovora* strain ATCC 49946. J. Bacteriol. 192, 2020–2021. 10.1128/JB.00022-10 20118253 PMC2838050

[B53] Shrestha R. Koo J. H. Park D. H. Hwang I. Hur J. H. Lim C. K. (2003). *Erwinia pyrifoliae*, a causal endemic pathogen of shoot blight of Asian pear tree in Korea. Plant Pathol. J. 19, 294–300. 10.5423/PPJ.2003.19.6.294

[B54] Smits T. H. M. Guerrero-Prieto V. M. Hernández-Escarcega G. Blom J. Goesmann A. Rezzonico F. (2014). Whole-Genome Sequencing of *Erwinia amylovora* strains from Mexico detects single nucleotide polymorphisms in rpsL conferring streptomycin resistance and in the avrRpt2 effector altering host interactions. Genome Announc. 2:e01229–13. 10.1128/genomeA.01229-13 24459281 PMC3900913

[B55] Smits T. H. M. Jaenicke S. Rezzonico F. Kamber T. Goesmann A. Frey J. E. (2010a). Complete genome sequence of the fire blight pathogen *Erwinia pyrifoliae* DSM 12163T and comparative genomic insights into plant pathogenicity. BMC Genomics 11:2. 10.1186/1471-2164-11-2 20047678 PMC2827408

[B56] Smits T. H. M. Rezzonico F. Kamber T. Blom J. Goesmann A. Frey J. E. (2010b). Complete genome sequence of the fire blight pathogen *Erwinia amylovora* CFBP1430 and comparison to other *Erwinia* spp. Mol. Plant Microbe Interact 23, 384–393. 10.1094/MPMI-23-4-0384 20192826

[B57] Smits T. H. M. Rezzonico F. Kamber T. Goesmann A. Ishimaru C. A. Stockwell V. O. (2010c). The genome sequence of the biocontrol agent *Pantoea vagans* strain C9-1. J. Bacteriol. 192, 6486–6487. 10.1128/JB.01122-10 20952567 PMC3008540

[B58] Smits T.H.M. Rezzonico F. Duffy B. (2011). Evolutionary insights from *Erwinia amylovora* genomics. J. Biotechnol. 155, 34–39. 10.1016/j.jbiotec.2010.10.075 21040749

[B59] Smits T. H. M. Rezzonico F. López M. M. Blom J. Goesmann A. Frey J. E. (2013). Phylogenetic position and virulence apparatus of the pear flower necrosis pathogen *Erwinia piriflorinigrans* CFBP 5888T as assessed by comparative genomics. Systematic Appl. Microbiol. 36, 449–456. 10.1016/j.syapm.2013.04.003 23726521

[B60] Sundin G. W. (2007). Genomic insights into the contribution of phytopathogenic bacterial plasmids to the evolutionary history of their hosts. Annu. Rev. Phytopathol. 45, 129–151. 10.1146/annurev.phyto.45.062806.094317 17367270

[B61] Sundin G. W. Bender C. L. (1995). Expression of the strA-strB streptomycin resistance genes in *Pseudomonas syringae* and *Xanthomonas campestris* and characterization of IS6100 in *X. campestris*. Appl. Environ. Microbiol. 61, 2891–2897. 7487022 10.1128/aem.61.8.2891-2897.1995 PMC167566

[B62] Sundin G. W. Bender C. L. (1996). Dissemination of the strA-strB streptomycin resistance genes among commensal and pathogenic bacteria from humans, animals, and plants. Mol. Ecol. 5, 133–143. 10.1111/j.1365-294X.1996.tb00299.x 9147689

[B63] Thapa S. P. Park D. H. Kim W. S. Choi B. S. Lim J. S. Choi I. Y. (2013). Comparative genomics of Japanese *Erwinia pyrifoliae* strain Ejp617 with closely related erwinias. Genome 56, 83–90. 10.1139/gen-2012-0094 23517317

[B64] Toth I. K. Pritchard L. Birch P. J. R. (2006). Comparative genomics reveals what makes an enterobacterial plant pathogen. Annu. Rev. Phytopathol. 44, 305–336. 10.1146/annurev.phyto.44.070505.143444 16704357

[B65] Triplett L. Zhao Y. F. Sundin G. W. (2006). Genetic differences among blight-causing *Erwinia* species with differing host specificities identified by suppression subtractive hybridization. Appl. Environ. Microbiol. 72, 7359–7364. 10.1128/AEM.01159-06 16963554 PMC1636173

[B66] Vrancken K. Holtappels M. Schoofs H. Deckers T. Valcke R. (2013). Pathogenicity and infection strategies of the fire blight pathogen *Erwinia amylovora* in Rosaceae: state of the art. Microbiology 159, 823–832. 10.1099/mic.0.064881-0 23493063

[B67] Wei Z. M. Beer S. V. (1993). HrpI of *Erwinia amylovora* functions in secretion of harpin and is a member of a new protein family. J Bacteriol. 175, 7958–7967. 8253684 10.1128/jb.175.24.7958-7967.1993 PMC206975

[B68] Young B. M. Young G. M. (2002). YplA is exported by the Ysc, Ysa, and flagellar type III secretion systems of *Yersinia enterocolitica*. J. Bacteriol. 184, 1324–1334. 10.1128/JB.184.5.1324-1334.2002 11844761 PMC134849

[B69] Zhao Y. F. Qi M. (2011). Comparative genomics of *Erwinia amylovora* and related *Erwinia* species—what do we learn? Genes 2, 627–639. 10.3390/genes2030627 24710213 PMC3927617

[B70] Zhao Y. F. Qi M. Wang D. (2011). Evolution and function of flagellar and non-flagellar type III secretion systems in *Erwinia amylovora*. Acta Hort. 896, 177–184. 10.17660/actahortic.2011.896.23

[B71] Zhao Y. F. Sundin G. W. Wang D. P. (2009). Construction and analysis of pathogenicity island deletion mutants in *Erwinia amylovora*. Can. J. Microbiol. 55, 457–464. 10.1139/W08-147 19396246

